# Phytochemical Composition of *Ziziphus lotus* (L.) Lam and Its Impact on the Metabolic Syndrome: A Review

**DOI:** 10.1155/adpp/8276090

**Published:** 2025-02-17

**Authors:** Chaimae Alla, Amanat Ali, Afaf Mehiou, Youssra Salhi, Nourelhouda Bouanani, Abdelkhaleq Legssyer, Abderrahim Ziyyat

**Affiliations:** Laboratory of Bioresources, Biotechnologies, Ethnopharmacology and Health, Department of Biology, Faculty of Sciences, University Mohammed First, Oujda, Morocco

**Keywords:** diabetes, hypertension, metabolic syndrome, obesity and dyslipidemia, polyphenols, *Ziziphus lotus* (L.)

## Abstract

The long-term pathological state known as metabolic syndrome is characterized by hypertension, insulin resistance diabetes, abdominal obesity, and hyperlipidemia. Seeking healthcare strategies with fewer side effects, such as herbal remedies, is preferable in terms of mitigating the negative consequences of synthetic medications. *Ziziphus lotus* (L.) (Rhamnaceae) or wild jujube, commonly known as “Sedra,” is one of the best choices as it contains a variety of phytochemicals and biologically active compounds. Several flavonoids and stilbenes have been recognized as the primary bioactive components in wild jujube, including rutin, hyperin, isoquercitrin, and resveratrol. These polyphenols are pharmacologically active and have broad-spectrum beneficial effects for reducing the risk factors associated with metabolic syndrome. They exhibit antioxidant and anti-inflammatory properties, regulate lipid metabolism, and possess antiobesity, antihypertensive, and antidiabetic characteristics. However, there are certain limitations to their therapeutic application, such as low bioavailability. Various strategies have been proposed to enhance their pharmacokinetic profile and therapeutic potential for future use. The main goal of this review is to explore the underlying mechanisms related to the therapeutic effects of wild jujube and its active compounds in the treatment and prevention of metabolic syndrome.

## 1. Introduction

The World Health Organization defines metabolic syndrome as a chronic pathological state. It features abdominal obesity, insulin resistance diabetes, hypertension, and hyperlipidemia [1]. The pathogenesis of metabolic syndrome results from the interplay of biological mechanisms such as low-grade chronic inflammation, oxidative stress, and hormonal imbalances. A key feature of the syndrome is insulin resistance. It is due to impaired signaling pathways in adipose tissue, skeletal muscle, and the liver. This predisposes the individual to hyperglycemia and dyslipidemia [2, 3]. Additionally, oxidative stress and inflammation may further drive metabolic syndrome by promoting endothelial dysfunction and thereby increasing cardiovascular risks [4].

The likelihood of metabolic syndrome increases due to several risk factors, such as an unhealthy diet, sedentarism, and environmental variables. Consequently, the management of metabolic syndrome needs to be multifactorial, rather than solely relying on clinical therapy at the time of diagnosis. These measures include adherence to a healthy lifestyle through balanced dietary habits, regular physical activity, and stress management [5]. Functional foods such as fiber, polyphenols, and omega-3 fatty acids have also showed preventive effects [6].

In recent years, the anticipated harmful side effects of synthetic medicines have drawn increasing attention to harness the potential of medicinal plants and natural phytochemicals in restoring metabolic balance [7]. Medicinal plants contain bioactive compounds such as flavonoids, anthocyanins, and phenolic acids that have showed potential against the symptoms of metabolic syndrome through mechanisms involving the reduction of oxidative stress, increase in insulin sensitivity, and modulation of inflammatory pathways and lipids metabolism. Flavonoids constitute a significant proportion of these bioactive chemicals [8]. Concurrently, plant extracts, foods rich in polyphenols, and pure polyphenols are being researched for their potential to prevent and/or treat disorders linked to metabolic syndrome [8]. For instance, 6-gingerol, a phenolic compound extracted from ginger, has been reported to effectively regulate AMP-activated protein kinase (AMPK) to enhance insulin sensitivity and modulate peroxisome proliferator–activated receptors (PPARs) involved in lipid metabolism [9]. Wild jujube (*Ziziphus lotus* [L.] [*Z. lotus*], Rhamnaceae) is one of the most popular herbal remedies used in folk medicine to cure and prevent a wide range of illnesses, including the risk factors of metabolic syndrome [10, 11]. Numerous phytochemicals and biologically active compounds have been identified in *Z*. *lotus* [12]. Among them, flavonoid and nonflavonoid polyphenols such as rutin, isoquercitrin, hyperin, and resveratrol are considered the primary bioactive components in wild jujube [13–15]. They have demonstrated a wide range of pharmacological advantages, such as improved lipid profiles [16], increased blood glucose tolerance, and better regulation of inflammatory markers [17]. Likewise, *Moringa oleifera*, a therapeutic plant widely investigated for its medicinal effects, has also showed benefits in the prevention of blood glucose spikes, blood pressure, hyperlipidemia, and obesity. Several molecular mechanisms of these actions have been elucidated, including AMPK activation, β-hydroxy-β-methylglutaryl-coenzyme A (HMG-CoA) inhibition, α-glucosidase inhibition, and glucose transporter type 4 (GLUT4) expression augmentation. These findings underscore the broader potential of medicinal plants to affect multiple pathways associated with metabolic syndrome [18].

This review encompasses the underlying mechanisms of action and focuses on the recent research regarding the therapeutic effects of wild jujube and its active constituents in the management and prevention of metabolic syndrome.

## 2. Methodology

A comprehensive literature review was undertaken using search terms such as “medicinal uses,” “phytochemistry,” “metabolic syndrome,” “diabetes,” “obesity,” “dyslipidemia,” and “hypertension” coupled with the core search keyword “*Z. lotus*.” All the citable data were gathered from various online databases such as Google Scholar, Science Direct, PubMed, Web of Science, and Scopus. Only articles published in English were included. Specific inclusion and exclusion criteria guided the selection of studies.

• The inclusion criteria include the following:
  - The literature documenting the traditional medicinal applications, therapeutic properties, or ethnopharmacological value of *Z. lotus*
  - The research detecting, purifying, or evaluating phytochemical components in *Z. lotus.*
  - The research on *Z. lotus* and its bioactive components
  - The research focusing on the components of metabolic syndrome, including diabetes, obesity, dyslipidemia, and hypertension
  - Peer-reviewed articles: both experimental studies (in vitro, animal, and clinical) and pertinent review articles.
• The exclusion criteria include the following:
  - Studies on other species of *Ziziphus* without relevant data on *Z. lotus*
  - Articles unrelated to metabolic syndrome or lacking experimental evidence
  - Non–peer-reviewed publications

The following data were extracted: All reported medicinal uses of *Z. lotus* in Africa and the Middle East; the phytochemical data, including compounds found in *Z. lotus*, the type of extract (i.e., aqueous, methanolic, and ethanolic) and the region where the specimens were collected; study design (animal model, human clinical trial, or in vitro), the dose of *Z. lotus* or its bioactive compounds, the bioactive compounds investigated, specific outcomes (such as blood glucose reduction, lipid profile improvements, or anti-inflammatory effects), and proposed mechanism of action with an emphasis on molecular targets.

The integrated molecular drawing platform ChemDraw 15.0 (PerkinElmer, Waltham, MA, USA) was used to sketch the chemical structures.

## 3. Botanical Description of *Z. lotus* (L)

**Figure 1:**
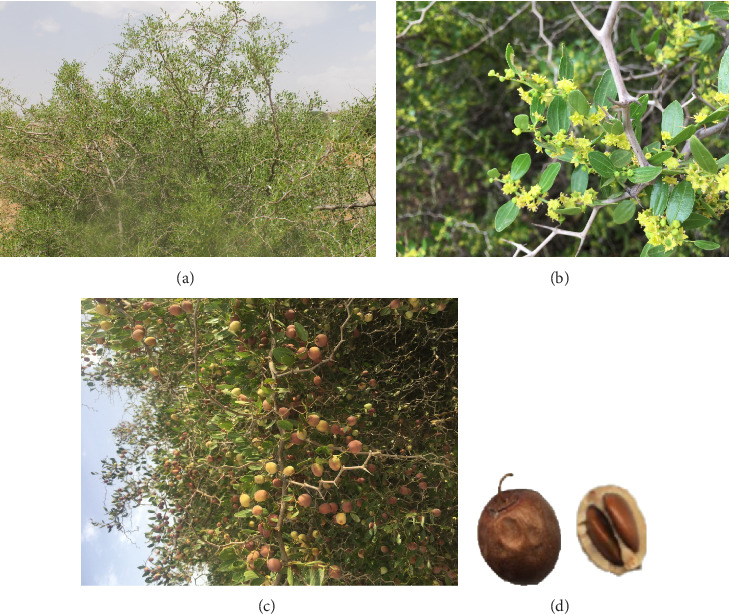
The whole plant (a); zigzag branches, leaves, and flowers (b); fruits in the tree (c); and isolated fruit and seeds (d) of *Z. lotus* (L.). Photos (a), (b), and (c) were taken by Pr Elachouri Mostafa in the region of Abbou Lakhal near Figuig City (Morocco) and (d) by Alla Chaimae.

*Z. lotus* is a thorny and frutescent shrub, reaching up to 2-3 m in height m in height (Figure 1(a)). The stems are glabrous and branching, with long woody shoots that sprout in short green branches. These branches begin as herbaceous and then become woody and bare when their leaves fall off. The young branches are yellowish-green, longitudinally striated, and have internodes of about 1.5–3 cm. They are characterized by a zigzag branching pattern, resembling a sympodial structure (Figure 1(b)). The leaves measure 1-2 cm long and 7–9 mm wide. They are simple, ovate-oblong, obtuse, alternate, crenulated, glabrous, and shortly petiolate, with two prickly and pointed stipules, out of which one is shorter and curved (Figure 1(b)). The leaves have a shiny and bright green lamina, with a three-nerved stretch from the base to the upper margin. They are covered with a thin cuticle, possess an astringent flavor, and are odorless. These leaves fall in the autumn and re-emerge in spring [19–22].

The inflorescence of the *Z. lotus* consists of a fascicle of 6–11 flowers; however, in most cases, only one fruit matures per inflorescence. The flowers are tiny and have a glabrous appearance throughout. The corolla is alternipetalous and yellowish, comprising five small petals. The calyx starts out green and gradually changes to yellow. The androecium's stamens are epipetalous, each with two styles, intrastaminal and joined by a disc. They also exhibit extensive dehiscence. The flowers are polygamous and mostly pentamerous (Figure 1(b)). The plant has an inferior ovary with two or three carpels that split at the stigma level. It contains two anatropous and integument ovules, distinguishing the gynoecium. The fruit ranges from yellow-orange to red in color (Figure 1(c)). At the ripened stage, it resembles an edible brown globular drupe with a diameter of 1–1.5 cm and can contain two round seeds (Figure 1(d)) [20, 22, 23].

## 4. Ecogeographical Distribution of *Z*. *lotus* (L.)

*Z*. *lotus* has a wide geographical and ecological range, highlighting its adaptability to diverse environmental conditions. It can be found in humid, subhumid, arid, semiarid, and Saharan climates [24], although it is more commonly present in dry and semiarid areas [25, 26]. This plant species thrives better in deep sandy soils [27, 28]. *Z*. *lotus* has a broad distribution in the Mediterranean region, especially in northern African countries such as Morocco, Algeria, Tunisia, Libya, and Egypt, as well as in some of northern European countries such as Spain, Cyprus, Sicily, and Greece [29]. Additionally, it has re-emerged in Yemen, on the island of Socotra, and across the Middle East in Palestine, Syria, and Türkiye [20, 24, 30, 31]. It is also grown in several Asian countries such as Iran, China, and South Korea [32].

## 5. Ethnomedicinal Uses of *Z*. *lotus* (L.)

*Z. lotus* (L.), commonly known as “Sedra,” is an important medicinal plant native to the arid Mediterranean regions [33]. It is used for the prevention and treatment of various ailments [12].

**Figure 2:**
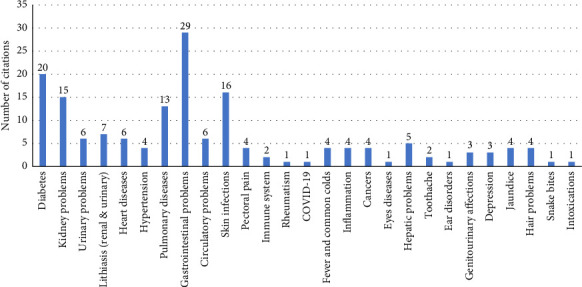
Number of citations for ailments treated with *Z. lotus* (L.) in ethnobotanical studies.

Based on the ethnobotanical surveys of medicinal plants conducted in Africa and the Middle East, 71 studies citing *Z*. *lotus* are reviewed and summarized in Table 1 and Figure 2. The countries included are Morocco, Algeria, Cameroon, Nigeria, Mauritania, Mauritius, Jordan, and Palestine.

Figure 2 displays the number of citations from ethnobotanical studies investigating the use of *Z. lotus* to treat various ailments. It highlights which health conditions have the most extensive medicinal use of *Z. lotus*.

**Figure 3:**
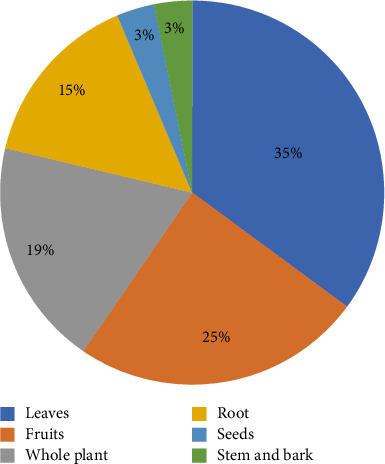
Percentage of plant parts used for ethnomedicinal purposes.

After analyzing the selected studies, it has been indicated that *Z. lotus* is extensively used in the treatment and control of diverse medical disorders such as gastrointestinal afflictions, diabetes, dermatological infections, renal dysfunctions, and respiratory ailments [10, 36, 37, 46, 100]. The indigenous medicinal applications of different parts of the plant are listed in Table 1. The leaves and fruits are the most used parts (35% and 25%, respectively), followed by whole plant (19%), roots (15%), barks (3%), and stems (3%) (Figure 3). Specifically, leaves are typically applied to treat conditions such as hypertension, diabetes, gastrointestinal problems, urinary tract issues, and skin infections [10, 46–63], whereas fruits are employed to relieve respiratory conditions, skin ailments, and digestive infections [83, 89, 95].

## 6. Phytochemistry of *Z*. *lotus* (L.)

### 6.1. Flavonoids

*Z. lotus* contains various parts, including leaves, branches, seeds, and root bark, that are major sources of flavonoid compounds contributing to its medicinal properties. A summary of these flavonoids is presented in Table 2.

Several reports have identified that *Z. lotus* contains substantial amounts of flavonoids, with primary emphasis on flavonols represented by kaempferol, quercetin, and myricetin. Flavonol glycosides have been ascertained as the main compounds identified in leaves and branch extracts (both aqueous and hydroethanolic). These include myricetin rutinoside, glycoside quercetin derivatives such as quercetin 3-*O*-rutinoside (rutin), quercetin-3-β-*D*-glucoside (isoquercitrin), quercetin-3-galactoside (hyperoside or hyperin), quercetin-3-*O*-(2,6-di-*O*-rhamnosyl-glucoside)-7-*O*-rhamnoside, quercetin-3-*O*-(2,6-di-*O*-rhamnosyl-glucoside), and quercetin-3-*O*-(2,6-di-*O*-rhamnosyl-glucoside)-7-*O*-glucuronide. Similarly, the glycoside kaempferol derivatives, including kaempferol-3-*O*-(2,6-di-*O*-rhamnosyl-glucoside), kaempferol-*O*-hexoside, kaempferol-3-*O*-rutinoside (nicotiflorin), and kaempferol-3-*O*-(6-*O*-rhamnosyl-glucoside). On the other hand, dihydrochalcones (phloretin: phloretin-di-*C*-hexoside), flavones (apigenin) and its glycoside derivatives (apigenin-*O*-hexoside-*O*-deoxyhexoside), flavan-3-ols (catechin, epicatechin, and epicatechin gallate), and naringin have also been found in leaves and root bark extracts, both in aqueous and hydroethanolic form from plants originating in Algeria and Morocco [13, 14, 102–105]. The biochemical profile of fruit's aqueous and hydroethanolic extracts from Tunisia showed the presence of several flavonoids, for instance, flavonols: resveratrol, isorhamnetin, quercetin 3-*O*-rutinoside (rutin), and hyperoside; flavones: luteolin-7-*O*-glucoside, apigenin 7-*O*-glucoside, apigenin, luteolin, diosmin, and cirsiliol; 3 proanthocyanidins (catechin); 1 chalcone (phloretin-3′,5′-di-glucoside); and flavanones: naringenin, eriodictyol moiety, and its glucoside derivatives, notably eriodictyol glycoside derivatives: eriodictyol-*O*-hexoside, eriodictyol-*O*-pentoside, and eriodictyol-*O*-deoxyhexoside [14, 15, 105–107]. On the other hand, the hydromethanolic extracts from the Moroccan plant included more quercetin 3-*O*-rhamnoside-7-*O*-glucoside [111]. In the flavonoids profile of aerial parts from Tunisia, quercetin-3-*O*-galactoside, quercetin-3-*O*-rhamnoside, and rutin were detected to be the most prevalent components in acetonic and hydroethanolic extracts [108, 109]. Furthermore, certain flavonoids were characterized in the ethanolic extract of seeds from Algeria. These compounds are referred to as biflavonoids, which include chamaejasmin; flavanones such as glucoliquiritin apioside, 5,6,7,8,3′,4′,5′-heptamethoxy flavanone, and robustaside D as an isoflavanone; and flavones: luteolin 7-(6′‴-acetylallosyl-(1- > 3)-glucosyl-(1- > 2)-glucoside, and apigenin 7-methyl ether 5-(6′-malonylglucoside). Kaempferol-3-*O*-robinobioside and gallocatechin were the only flavonols and flavan-3-ols identified, respectively [110].

### 6.2. Phenolic Acids

The identification of various bioactive compounds in different extracts of *Z. lotus* highlights its medicinal potential, particularly in the treatment of a wide range of ailments. The analysis of ethanolic and hydroethanolic fruit extracts from Tunisia, Morocco, and Algeria showed higher concentrations of phenolic acids compared to the methanolic extracts, such as *p*-hydroxybenzoic acid, *p*-coumaric acid, benzoic acid, sinapic acid, *p*-coumaroyl glucose, cinnamic acid derivatives, galloyl shikimic acid, trans-ferulic acid, rosmarinic acid, syringic acid, protocatechuic acid, gallic acid, quinic acid, and caffeic acid derivatives [15, 106, 111, 112]. In addition, two primary phenolic acids have also been revealed in the methanolic leaf extract from Algeria and Morocco *Z. lotus*, including fumaric acid and gentisic acid, whereas gallic acid, chlorogenic acid, caffeic acid, vanillic acid, *p*-coumaric acid, benzoic acid, and *p*-hydroxybenzoic acid have all been found to be present in the water extract [13, 14, 113].

In addition to the aforementioned nonflavonoid polyphenols, the fruits from Morocco also possess another prominent compound “resveratrol,” which belongs to the stilbene category [14]. A summary of the identified phenolic acids is provided in Table 2.

### 6.3. Alkaloids

Alkaloids are well known for their therapeutic potential in managing various aspects including obesity, insulin resistance, and hypertension [121]. Several cyclopeptide alkaloids were recovered from *Z. lotus* root bark of Tunisian provenance, such as lotusine A, B, C, D, E, F, and G [114–116]. The other cyclopeptide alkaloids that have been identified in aerial parts were named lotusanine A and lotusanine B. These were isolated along with sanjoinenine, sanjoinine F, and frangufoline [117]. The alkaloid content in Tunisian *Z. lotus* was found to be lower than that of polyphenol compounds [109]. A summary of these alkaloids, along with their presence in different plant parts, is provided in Table 2.

### 6.4. Saponins

Saponins have showed promise in modulating lipid metabolism and improving insulin sensitivity [122]. It is worth mentioning that all parts of *Z. lotus* contain saponins, but higher contents were detected in the leaves (340 mg/100 g) [120]. Indeed, according to Renault et al. [119] and Maciuk et al. [118], numerous saponins belonging to the dammarane class have been identified in leaves, including jujuboside B, jujuba saponin IV, and three jujubogenin glycosides. Additionally, the root bark yielded four saponins of the same class, specifically jujuboside A, jujuboside C, lotoside I, and lotoside II [118, 119]. The summary of these saponins across the plant's parts can be found in Table 2.

### 6.5. Other Bioactive Compounds

In addition to alkaloids and saponins, *Z. lotus* contains a variety of bioactive compounds such as condensed tannins, secoiridoids, fatty acids, and vitamins, which have potential health benefits [25]. *Z. lotus* from Algeria and Morocco also contains condensed tannins, which were found to be present in the highest concentrations in the aqueous extract and the lowest in the petroleum ether extract [104, 123]. Higher levels of condensed tannins were observed in leaves [107, 124, 125]. Secoiridoids were first found in branches and then in leaves, with oleoside being the most abundant compound in branches and oleuropein in leaves from Algeria [105]. The richest sources of fatty acids in the fruit were in its aqueous and methanolic extracts, as well as in the seed oil. The principal substances among them included oleic acid, linoleic acid, and elaidic acid [125–131]. Different parts of *Z. lotus* have also been reported to contain some vitamins such as C, A, and E; carotenoids; β-tocopherol; and *δ*-tocopherol [127, 128, 132].

### 6.6. Volatile Compounds

According to our knowledge, there is limited research on the essential oil analysis of *Z. lotus*. Bekkar et al. were the first to focus on the essential oils of Algerian *Z. lotus* leaves and reported the presence of abundant amounts of phthalate esters and terpenes. Diisooctyl phthalate was identified as the main compound (89.85%), followed by linalool (2.14%). Minor quantities of other bioactive components have also been identified in this plant, for example, thymol (0.56%), *p*-cymene (0.35%), caryophyllene (0.26%), 2-undecanone (0.20%), ɤ-terpinene (0.16%), linalyl acetate (0.14%), and methyl eugenol + 97 ION (0.13%). Due to its dominance, this essential oil has been categorized as a diisooctyl phthalate chemotype [133]. During the analysis of fruit essential oils from Algerian *Z. lotus*, some hydrocarbons were also identified, such as heptacosane (3.7%) and nonacosane (3.7%). They also detected sesquiterpenes such as α- and β-eudesmol [130].

A comprehensive study conducted by Letaief et al. in Tunisia unveiled the presence of various compounds in the essential oil extracted from the aerial parts and fruits of the plant. The analysis revealed the presence of significant amounts of sesquiterpenes in the aerial parts, including hexahydro-farnesyl acetone (23.2%), E-nerolidol (5.9%), α-calacorene (3%), and D-nerolidol (1.9%). Additionally, apocarotenoid derivatives such as geranylacetone (12.5%), trans-β-ionone (6%), 2-undecanone (2.9%), and damascenone (1.3%) were also identified. The essential oil extracted from the fruits predominantly contained an oxygenated sesquiterpene along with 2-pentadecanone (16.9%) [134].

## 7. Data Related to the Safety of *Z*. *lotus* (L.)

There is a common fallacy that herbal medicines are safe and do not cause any adverse reactions. No reliable scientific data are available about their safety, as some of them can result in potential adverse effects leading to injuries and life-threatening conditions [135]. Acute toxicity tests were performed on rats to examine the impact of a water extract of Tunisian *Z*. *lotus* root bark extracts on mice. The results indicated that intraperitoneal administration of various dosages (50–1000 mg/kg body weight [BW]) produced variable results. No mortality was seen at 50 mg/kg BW, whereas a dose level of 1000 mg/kg BW provoked 100% mortality in mice. The median lethal dose (LD50) value was determined at 400 mg/kg BW [136]. Similarly, the results from another study showed that the oral administration of different doses of aqueous extracts of Moroccan *Z. lotus* fruit (200–2000 mg/kg BW) did not result in any mortality or behavioral changes in the rats [137]. On the contrary, no morbidity or distinctive clinical signs were recorded during the observation period after the administration of a single dose of 5000 mg/kg BW of the seed oil in mice [138]. For Algerian *Z. lotus*, the leaf essential oil given at a dose of 5000 mg/kg BW did not produce any visible toxic effects; therefore, LD50 seemed to be greater than 5000 mg/kg BW [133]. On the other hand, it has been demonstrated that the aqueous extract obtained from the leaves, fed at 2000 mg/kg BW as a single dose, did neither cause any mortality nor apparent toxicity symptoms in rats. It was concluded that the LD50 value appeared to be more than 2000 mg/kg BW [139].

## 8. *Z*. *lotus* (L.) Extracts and the Prevention of Metabolic Syndrome

According to the National Cholesterol Education Program's (NCEP ATP III) definition, metabolic syndrome is suspected if three or more of the following five criteria are met: blood pressure greater than 130/85 mmHg, fasting triglyceride (TG) level greater than 40 mg/dL, fasting high-density lipoprotein (HDL-C) cholesterol level less than 40 mg/dL (for men) or 50 mg/dL (for women), fasting blood sugar level greater than 100 mg/dL, and waist circumference exceeding 102 cm for men or 88 cm for women [140]. The development of metabolic syndrome is influenced by many factors, including lifestyle, insulin resistance, genetic predisposition, being overweight or obese, and the natural process of aging. Many risk factors associated with metabolic syndrome may not display symptoms or signs, except for having a waist circumference [141].

The study investigating the antidiabetic and antihyperlipidemic activities of *Z. lotus* has showed that the aqueous extract from both fruits and leaves exhibited the greatest in *vitro* inhibitory impact against α-glucosidase (half-maximal inhibitory concentration (IC_50_): 8.66–27.95 μg/mL) and α-amylase (IC_50_: 20.40–31.91 μg/mL) compared to standard acarbose [14]. Furthermore, the aqueous extract of the fruit sustained average blood glucose levels, leading to a substantial attenuation in TG and total cholesterol (TC) and causing a rise in HDL-C in mice receiving a high-fat diet compared to those on a normal diet [142]. At dosages of 200 and 400 mg/kg BW, this extract reduced the atherogenic index and the TC/HDL-C ratio, effectively preventing atherosclerosis, which is directly linked to cardiovascular disease [143, 144]. The aqueous extract also significantly lowered the plasma levels of proinflammatory markers, including interleukin-6 (IL-6) and tumor necrosis factor-alpha (TNF-α), as well as monocyte chemoattractant protein-1 (MCP-1) mRNA expression, and produced higher levels of anti-inflammatory cytokine IL-10 in hepatic and adipose tissues. These changes have shown to affect insulin signaling during obesity [145]. In addition to improving glucose tolerance and reducing insulin resistance in the homeostatic model, *Z*. *lotus* fruit powder improved the levels of circulating glucose and insulin in HFD-induced obesity in C57BL/6j mice. The expression of hepatic glucose-6-phosphatase (G-6-Pase) mRNA was downregulated, inhibiting the liver's ability to produce glucose. As evidenced by a decline in fatty acid synthase (FAS) and acetyl-CoA carboxylase 1 (ACC1) mRNA expression, *Z. lotus* fruit was found to lower lipogenesis [146]. Besides raising the PPARγ and carnitine palmitoyltransferase 1 alpha mRNA expression, which were lowered in obese mice, *Z. lotus* fruit appeared to increase the use of fatty acids as an energy source [147].

According to a study conducted by Benammar et al. in 2014, it was observed that the aqueous extracts obtained from the roots and leaves exhibited a substantial decline in blood glucose levels in streptozotocin (STZ)-induced type 2 diabetic rats. These extracts were also shown to expedite the recovery of glucose levels during the glucose tolerance test. Furthermore, as demonstrated by the measures of kinetics of red blood lysis and oxygen radical absorbance capacity, it was noticed that these extracts increased the antioxidant status in diabetic rats. Catalase and glutathione peroxidase activities were greatly enhanced, while glutathione reductase activity was restored [148].

## 9. The Bioactive Compounds of *Z*. *lotus* (L.) and Their Beneficial Effects on Metabolic Syndrome

Bioactive compounds are naturally occurring substances in foods that positively affect health. These substances can lower the chances of developing metabolic syndrome by positively affecting BW, blood pressure, blood glucose control, endothelial function, lipid profile, inflammation, and oxidative stress [149]. *Z*. *lotus* contains elements such as polyphenols, which actively participate in preventing the onset of metabolic syndrome. They exhibit anti-inflammatory and antioxidative properties and manage the oxidative stress linked to metabolic syndrome [25, 150]. The research indicates that the compounds found in *Z*. *lotus* can improve insulin sensitivity and regulate glucose metabolism [150, 151]. They also contribute to reducing adiposity by inhibiting adipogenesis which is crucial for addressing obesity [152]. The targeted effects of compounds derived from *Z*. *lotus* demonstrate their potential in preventing and managing the health conditions associated with metabolic syndrome [25].  Table 3 summarizes the main mechanisms.

### 9.1. Flavonoids and Nonflavonoid Polyphenols

**Figure 4:**
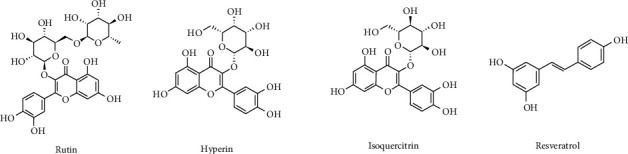
Chemical structures of *Z*. *lotus* (L.) main flavonoids and stilbene.

**Figure 5:**
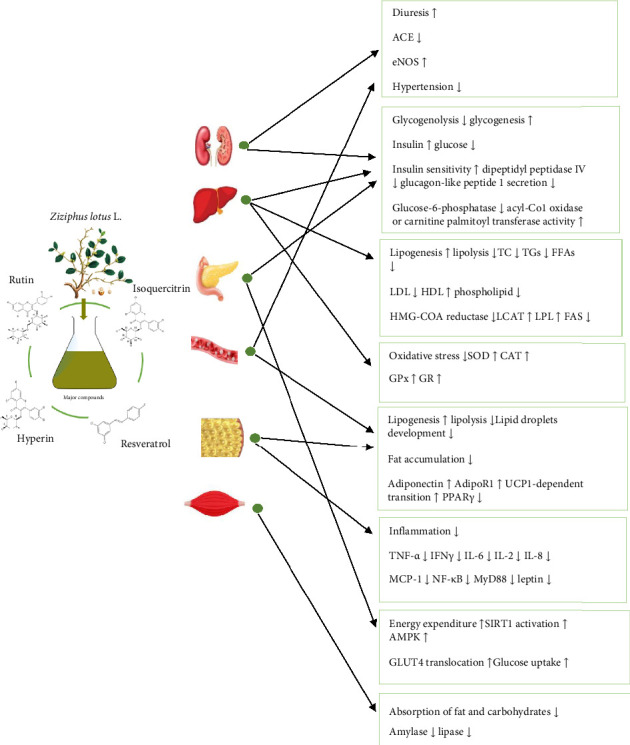
Schematic diagram depicting the various mechanisms employed by bioactive compounds of *Z. lotus* (L.) in managing the metabolic syndrome. Increase (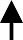) or decrease (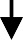) after bioactive compounds supplementation.

Many flavonoids and nonflavonoid polyphenols demonstrate favorable effects in mitigating the risk factors associated with metabolic syndrome [153–155]. In this respect, our attention has been devoted to three flavonoids and one nonflavonoid (stilbene) polyphenol, the most abundant chemical substances found in the plant. In particular, the prominent polyphenols encompass rutin, hyperin, isoquercitrin, and resveratrol (Figure 4) [13–15], which are effective in improving conditions such as obesity, diabetes, hypertension, and dyslipidemia (Figure 5).

#### 9.1.1. Effects on Diabetes

Rutin, isoquercitrin, hyperin, and resveratrol are the bioactive compounds that have been mostly studied for their potential to reduce the risk of diabetes.

##### 9.1.1.1. Lowering Blood Glucose Levels

Rutin at doses of 50 or 100 mg/kg BW has shown to effectively lower the fasting blood glucose (FBG) and glycated hemoglobin (HbA1c) levels in STZ-induced type 2 diabetic rat models [156–158]. Isoquercitrin is also stated to reduce FBG levels in STZ-induced type 2 diabetic mice [159]. The expression of glucose transporter Type 2 was also found to be dramatically upregulated by isoquercitrin. In addition, the key glucose-regulating metabolic enzymes, such as glucose-6 phosphate dehydrogenase and phosphoenolpyruvate carboxykinase, were also well controlled [160]. Hyperin significantly reduces glycemia levels, protects the pancreatic beta-cells, and enhances insulin secretion in diabetic mice caused by an HFD/alloxan [161, 162]. Hyperin has showed to play a significant role in controlling blood glucose levels by improving the functions of pancreatic islets in rats. At doses of 25 and 50 mg/kg BW in STZ-induced type 2 diabetic rats, it significantly reduced the blood glucose levels after 120 min by increasing glycolysis and decreasing gluconeogenesis. These effects are comparable to the standard medication glibenclamide used for treating type 2 diabetes [163]. Resveratrol dramatically alleviates hyperglycemia and has a beneficial impact on insulin sensitivity in diabetes animal models generated by STZ or alloxan injection [164].

##### 9.1.1.2. Improving the Insulin Sensitivity

Through stimulating the insulin receptor (IR) kinase activity, rutin can act as a glycemic control agent by activating the insulin signaling pathway, thereby increasing GLUT4 translocation and glucose uptake [165]. It also enhances the expression of PPARγ, improves glucose uptake by adipose tissues and skeletal muscles, and reduces insulin resistance [166]. Moreover, rutin can boost the insulin release triggered by hyperglycemia in rat beta-cells while preserving their capacity to sense glucose under high glucose conditions [167]. Rutin has also been reported to reduce insulin resistance by enhancing glucose uptake in insulin-resistant cells in FL83B hepatocytes exposed to high glucose levels and improving insulin signaling via phosphorylated protein kinase C activity suppression [168]. Isoquercitrin positively impacts insulin sensitivity by targeting dipeptidyl peptidase IV, with an IC_50_ value of 44.93 μg/mL [159]. Furthermore, in the HFD mice model, quercetin and its glycosides such as isoquercitrin averted insulin resistance by improving GLUT4 translocation in skeletal muscles via modulating AMPK-driven pathways [169]. The research conducted by Jayachandran et al. [160] showed that when STZ-induced diabetic rats were supplemented with isoquercitrin; it regulated the expression of genes involved in insulin signaling, such as insulin, IR, IRS-1, IRS-2, and protein kinase B (PKB). In human liver cancer cell line (HepG2) cells resistant to insulin, hyperin enhances the expression of the PPARγ protein [170]. Resveratrol has shown to improve FBG, insulin sensitivity, HbA1c, and homeostasis model assessment of insulin resistance values in participants with type 2 diabetes mellitus and obesity [171–173]. It has also shown to upregulate the expression of activated Sirtuin 1 (SIRT1) and AMPK in skeletal muscles [174]. It also stimulates insulin production and improves skeletal muscles glucose absorption via the phosphoinositide 3-kinase—PKB signaling pathway. Likewise, it leads to a rise in the expression of GLUT4 in skeletal muscles and a decline in phosphoenolpyruvate carboxy kinase in the liver [175].

##### 9.1.1.3. Inhibition of Enzymes Associated With Type 2 Diabetes

Rutin exhibits a potent inhibitory impact on the main enzymes associated with type 2 diabetes (α-amylase and α-glucosidase), with IC_50_ values of 26.27 and 22.61 μg/mL, respectively [176]. Isoquercitrin potently inhibits the α-glucosidase, with an IC_50_ of 85.82 μg/mL [177]. Hyperin shows a significant α-amylase inhibition effect, with an IC_50_ value of 491 μg/mL [178]. Resveratrol inhibits the α-glucosidase activity, and its ability is 2.5 times greater than that of acarbose (IC_50_ = 91 μg/mL) [179].

##### 9.1.1.4. Reducing Oxidative Stress and Inflammation

Rutin reduces G-6-Pase, a crucial gluconeogenic enzyme, in the liver and kidney of diabetic rats by 31% and 37%, respectively [157, 180]. The protective effects of hyperin are associated with the recovery of intracellular redox equilibrium and mitochondrial function via the suppression of mitogen-activated protein kinase signaling, caspase-dependent apoptosis, calcium release, and thioredoxin-interacting protein expression [162]. Hyperin eliminates hyperglycemia-induced vascular inflammation by suppressing monocyte adhesion, cell adhesion molecule expression, reactive oxygen species formation, and nuclear factor (NF-κB) activation [181]. Resveratrol at 5 mg/kg BW can avert the hyperglycemia-induced oxidative stress by boosting oxidative markers such as the lipid peroxidation index and nitrite/nitrate content, and by decreasing the glutathione ratio. It also boosts the enzymic antioxidants such as superoxide dismutase in hepatic tissues. The levels of hepatic proinflammatory cytokines TNF-α and IL-6 are substantially reduced [182, 183]. Resveratrol pretreatment has showed to impede the caspase-3 and poly(ADP-ribose) polymerase (PARP) activation by STZ injection, thus preventing pancreatic beta-cell apoptosis [184].

#### 9.1.2. Effects on Obesity

##### 9.1.2.1. Inflammation

Rutin has been suggested to reduce the TNF-α and interferon-gamma (IFNγ) expression, thereby shielding the liver from excess fat-induced inflammation [185]. Additionally, rutin has been reported to suppress the expression of proinflammatory markers, such as TNF-α, MCP-1, IFNγ, IL-6, IL-2, NF-κB, myeloid differentiation primary response gene 88, and lipopolysaccharide-binding protein in adipose tissues [186, 187]. Isoquercitrin, on the other hand, modulated the elevated proinflammatory secretion induced by obesity in HFD mice, by affecting adipokine expression (decreased leptin and increased adiponectin levels) [188]. It also influences adipokine expression and attenuates NF-κB and TNF-α expression in the adipose tissue and liver in HFD + STZ rats [189]. Adipocytes pretreated with resveratrol demonstrated an anti-inflammatory action, as evidenced by limiting the release of TNF-α and IL-6 and the activation of inflammatory proteins, such as extracellular receptor–activated kinase (ERK) and NF-κB [190]. Furthermore, inflammatory-related adipokines such as leptin, IL-6, IL-8, and MCP-1 were all downregulated by resveratrol in human adipose tissues [191, 192].

##### 9.1.2.2. Impact on Metabolism and Energy Expenditure

Rutin has the potential to ameliorate obesity by promoting the formation of brown-like adipocytes in subcutaneous adipose tissues and by raising brown adipose tissue (BAT) activity. It also inhibits the liver's ability to produce fatty acids by repressing Srebp1c and Cd36 transcription, while enhancing the expression of PPAR gamma coactivator 1 (Pgc1) and deiodinase iodothyronine type 2 (Dio2), two genes involved in energy expenditure, in BAT [193]. Both genetically obese (Db/Db) and diet-induced obese mice showed significant reductions in adiposity, increased energy expenditure, and improved glucose homeostasis after rutin treatment. In obesity mouse models, rutin-induced brown adipocyte (beige) formation [193]. Isoquercitrin prevented weight gain and mesenteric adipose tissue fat deposition. In addition, it stimulated lipogenesis while suppressing lipolysis in white adipose tissues and liver [194]. It also inhibited the formation of cellular lipid droplets by activating AMP kinase, a crucial cellular energy sensor, and its dependent signaling pathways. These pathways improved the expression levels of adiponectin receptor 1 while reducing the activity of sterol regulatory element-binding transcription factor 1 (SREBP-1) and the expression of lipogenic genes/FAS signals [194, 195]. Hyperin possesses the ability to prevent the Swiss 3T3 cell line, preadipocyte clone 1 (3T3-L1) preadipocytes from maturing into fully developed adipocytes. By decreasing PPAR, CCAAT/enhancer-binding proteins (C/EBP), and SREBP-1c gene expression, as well as that of lipoprotein lipase (LPL), hyperin also decreased the intracellular accumulation of TG in mature adipocytes. Even at modest doses, it has showed to effectively prevent adipogenesis [196]. Through cyclin-dependent kinase 6 (CDK6) and transcription factor EB (TFEB), (CDK6-TFEB) signaling, hyperin at 80 mg/kg BW has shown to resist obesity by stimulating the uncoupling protein 1 (UCP1)-dependent transition of white to beige fat, aiding in lipophagy, a particular form of autophagy that helps in breaking down lipid droplets, and enhancing lipolysis [197]. Resveratrol promotes the expression of SIRT1, a key target of cellular energy metabolism and mitochondrial homeostasis, resulting in a dose-dependent reduction in fat formation within cells. It can also lower the expression of adipogenesis-related genes and proteins, including PPAR, C/EBP, and SREBP-1c [198, 199]. The lipolytic action of human and rat adipocytes was boosted by resveratrol through β-adrenergic stimulation and the subsequent rise in cyclic adenosine monophosphate levels [200, 201]. However, by blocking the effects of insulin and insulin-like lipogenic agents, resveratrol can slow down the transfer of glucose into the adipocytes' fat stores [202].

##### 9.1.2.3. Fat Storage and Lipid Metabolism

Rutin treatment significantly decreased lipid droplet development in 3T3-L1 cells, promoted the expression of thermogenic markers, and lowered the expression of adipogenic genes [203]. Through inhibiting Wnt/catenin signaling, isoquercitrin restrains the 3T3-L1 cells from differentiating into adipocytes [204]. The antiobesity effects of resveratrol hint at the nonexpression of adipogenesis or lipogenesis genes such as PPAR-γ, ACC1, and FAS in visceral adipose tissues. Resveratrol has also showed to suppress the expression of LPL and stearoyl-CoA desaturase 1 fatty acid biosynthesis genes in the liver. However, as resveratrol regulates the gut microbiota, it may enhance the fasting-induced adipose factor (FIAF) gene's expression through the FIAF signaling pathway in the intestine [205].

#### 9.1.3. Effects on Dyslipidemia

##### 9.1.3.1. Lipid and Lipoprotein Modulation

Rutin supplementation was found to successfully lower the serum lipid levels in HFD and STZ-elicited diabetic rats and in hypertriacylglyceremic rats fed a high-fat and high-fructose diet including TC, TG, and low-density lipoprotein cholesterol (LDL) while achieving a considerable boost in HDL [206–208]. Isoquercitrin has also showed to reduce the plasma concentrations of C-peptide, TG, and TC at a dose of 200 mg/kg BW in KK-Ay mice, a model of spontaneous diabetes [209]. Additionally, plasma TG and TC levels were markedly lowered in obese mice produced by HFD after receiving isoquercitrin at 5 mg/kg BW [210]. Furthermore, TC, TG, LDL-C, VLDL-C, free fatty acids, and phospholipid levels were reduced following supplementation, whereas HDL-C levels increased [211, 212]. Administration of hyperin (200 mg/kg BW) in diabetic mice models produced by HFD or alloxan or STZ resulted in a significant reduction in dyslipidemia markers, including TG, TC, and LDL-C levels, whereas HDL-C levels were elevated [161, 163]. It has showed that hyperin significantly enhanced TG metabolism and reduced TG in insulin-resistant HepG2 cells at a modest concentration of 0.75 μg/mL [170]. Resveratrol significantly and dose-dependently alleviated the blood TC, LDL-C, TG levels, and the arteriosclerosis index in male Sprague–Dawley rats fed a hyperlipidemic diet for 4 weeks. Although HDL-C levels tended to rise, the hepatic concentrations of TC and TG were lowered [213]. Similar results were observed in hyperlipidemic mice treated for 6 weeks [214] and in obese rats fed for 8 weeks [215]. In individuals with dyslipidemia and type 2 diabetes, oral supplementation with resveratrol (100–250 mg/d) has showed to effectively improve glycemic control and can lower the HbA1c, TC, and TG concentrations [216].

##### 9.1.3.2. Regulation of Enzyme Activities

In diabetic rat models, rutin improved the LPL and lecithin cholesterol acyltransferase activities in plasma while showing a decreasing trend in (HMG Co-A) reductase activity [208]. Isoquercitrin treatment (40 mg/kg BW) considerably raised the levels of fat-metabolizing enzymes, such as LPL and lecithin cholesterol acyltransferase, while significantly lowering HMG Co-A levels [211, 212]. Hyperin showed 58.9% inhibition of HMG Co-A reductase [217]. Resveratrol has showed to downregulate the liver enzyme HMG-CoA and impact the expression of cholesterol metabolism-related enzymes [218, 219], with a simultaneous increase in the production and release of bile acids due to improvement of hepatic cholesterol 7a-hydroxylase expression [220].

##### 9.1.3.3. Impact on Gene Expression and Fatty Acid Metabolism

According to Park et al., rutin supplementation at 1000 mg/kg BW enhanced the fecal sterol excretion, thereby lessening dietary cholesterol absorption [221]. Due to its ability to control the expression of genes (in particular the cyclooxygenase 1.3 (cox-1.3), S-trimethylglycine dehydrogenase-3 (stdh-3), and fatty acid transporter 7 (fat-7)), and by extending the production of the related unsaturated fatty acid, rutin diminished fat accumulation in *Caenorhabditis elegans* [222]. Isoquercitrin is attributed to the effective control of mRNA expression of AMPK-α and ACC [211, 212]. Additionally, via an AMPK-dependent mechanism, resveratrol boosts the LDL receptor expression on hepatocytes and hepatic uptake of LDL [223, 224].

#### 9.1.4. Effects on Hypertension

##### 9.1.4.1. Inhibition of Angiotensin-Converting Enzyme (ACE)

Rutin supplementation has been reported to improve hypertension in rats fed a high-carbohydrate HFD as well as in Nω-nitro-L-arginine methyl ester (L-NAME)-induced hypertensive rats [225, 226]. Additionally, normotensive rats showed improvements at 1–3 mg/kg BW [227]. The antihypertensive impact of rutin is exerted through its capacity to block the ACE, with an inhibitory potency that reached 87% at 151.12 μg/mL with an IC_50_ value of 19.34 μg/mL [225, 228]. Groups of normotensive and spontaneously hypertensive rats (SHR) receiving intravenous doses of isoquercitrin (0.5–4 mg/kg BW) exhibited dose-dependent hypotension that relied on the inhibition of ACE activity (55% at 4 mg/kg BW), leading to an inhibition in angiotensin II (Ang-II) production in the serum [229]. ACE showed to be significantly inhibited by hyperin, with an IC_50_ value of 54.40 μg/mL [230].

##### 9.1.4.2. Promotion of Diuresis

Gasparotto Junior et al. explored the mechanisms underlying the hypotensive impact of isoquercitrin in SHR. They observed that the diuretic effect of isoquercitrin was dose-dependent (5–10 mg/kg BW in SHR) without any toxic symptoms. They reported that this effect is connected to its diuretic activity, by increasing the excretion of Na^+^ while leaving K^+^ levels unchanged in the urine [231]. In another study, the same authors discovered that the mechanism by which isoquercitrin (10 mg/kg; p.o.) increases diuresis is mostly connected to a higher bioavailability of bradykinin, prostaglandin I2, and nitric oxide, as well as the inhibitory effect on Na^+^/K^+^-ATPase [232]. The treatment with hyperin (6 mg/kg BW) lowered the high blood pressure in L-NAME-induced hypertensive rats. It has proposed that it was achieved by enhancing the diuretic and renal nitric oxide synthase (NOS) activities with a simultaneous reduction in the peripheral resistance through a decrease in the medial layer thickness of coronary vessels [233].

##### 9.1.4.3. Activation of the Nitric Oxide–Guanylyl Cyclase Pathway

In isolated rat aorta rings, rutin exhibited an endothelium-dependent vasorelaxant effect in a dose-dependent manner via the nitric oxide–guanylyl cyclase pathway, a prostaglandin-mediated mechanism through the activation of ATP-sensitive potassium channels [234]. Resistance arteries in the rat mesenteric vascular bed respond to isoquercitrin at concentrations of 64.44, 139.32, and 464.4 μg/mL by inducing both endothelium-dependent and endothelium-independent vasodilation, with a peak relaxation of 57.8% observed at 464.4 μg/mL. This relaxation was triggered by nonselective K_Ca_ (calcium-activated) channel activation, nitric oxide, and the production of other endothelium mediators mainly at high levels of isoquercitrin [235]. In isolated rat aorta, hyperin demonstrated endothelium-dependent vasodilatory action with an EC_50_ of 91.3 μg/mL. This effect may be due to the promotion of endogenous hydrogen sulfide in arterial endotheliocytes and the opening of K_Ca_ channels [236, 237]. The possible mechanisms of resveratrol's antihypertensive action have also been demonstrated, including endothelial NO generation dependent on calcium-eNOS activation [238]. In addition, the improved bioavailability of NO was correlated with vasodilatory activities brought about by resveratrol in SHRs and Ang-II mice [239]. The endothelial NO bioactivity was boosted by resveratrol via the stimulation of eNOS gene transcription and stability [240]. The suppression of elevated Giα protein levels and the upstream signaling molecules promoting Gi protein overexpression in vascular smooth muscle cells are thought to be responsible for the attenuation of hypertension by resveratrol [241].

In addition to these effects, in high-salt-induced hypertensive mice, hyperin caused a drop in blood pressure by suppressing chymase, an independent Ang-II-forming enzyme [242]. In a clinical investigation, distal segments of internal thoracic arteries from patients undergoing coronary revascularization were treated with isoquercitrin at 28 μg/mL. This treatment produced a potent vasorelaxant effect, reaching 53.29%, that was mediated by activating the COX pathway [243]. In human umbilical vein endothelial cells under high glucose stress, the proapoptotic proteins p53, Bax, and cleaved caspase-3 expression was considerably downregulated by isoquercitrin treatment, whereas the antiapoptotic protein B-cell lymphoma 2 was raised in a dose-dependent manner [244]. Rutin also reestablished vascular reactivity and baroreflex sensitivity in hypertensive rats [245]. Furthermore, under high glucose conditions, rutin restored the acetylcholine-mediated endothelium function in aortic tissues by substantially recovering NO generation and suppressing the expression of nicotinamide adenine dinucleotide phosphate oxidase (NADPH) 4 and nucleotide-binding oligomerization domain (NOD)-like receptor family pyrin domain containing three inflammasome pathways, both in vitro and in vivo [246]. A clinical trial carried out by Sattanathan et al. [247] involving patients with type 2 diabetes mellitus showed that rutin decreased both systolic and diastolic blood pressure.

One possible explanation could be that rutin, isoquercitrin, hyperin, and resveratrol could cooperate synergistically with each other, leading to better insulin sensitivity, less oxidative stress, and/or better lipid metabolism. As a result of their complementary antioxidant, anti-inflammatory, and vasodilatory properties, they may modulate important enzymes of glucose and lipid metabolism and NO synthesis more effectively, resulting in a greater therapeutic benefit for metabolic syndrome and cardiovascular risk factor management.

### 9.2. The Biosynthetic Pathways

Rutin, isoquercitrin, hyperin, and resveratrol share several common steps in their biosynthetic pathways, primarily originating in the phenylpropanoid pathway, starting from phenylalanine. Phenylalanine is first converted to trans-cinnamic acid via the action of phenylalanine ammonia-lyase. This intermediate is then hydroxylated by cinnamate-4-hydroxylase to generate *p*-coumaric acid, which is, in turn, activated by 4-coumarate-CoA ligase to yield 4-coumaroyl-CoA. The chalcone synthase enzyme catalyzes the condensation of 4-coumaroyl-CoA into chalcone, which is then converted to naringenin by chalcone isomerase. Flavanone-3-hydroxylase catalyzes the hydroxylation of the common precursor naringenin to dihydrokaempferol, which can then be converted into quercetin via flavonol synthase. Quercetin is glycosylated in the biosynthesis of rutin and isoquercitrin: Quercetin is rhamnosylated and glucosylated in rutin, while quercetin is glycosylated at the 3-*O* position in isoquercitrin by UDP-glucose: flavonoid 3-*O*-glucosyltransferase (UGT) [248, 249]. In turn, hyperin is an *O*-glycoside of quercetin-bearing a galactopyranoside moiety, formed by the action of sugar transferases, acting through with specificity in favor of galactose [250]. Conversely, resveratrol is produced via a branch of the phenylpropanoid pathway, where stilbene synthase catalyzes the condensation between *p*-coumaroyl-CoA and three malonyl-CoA molecules leading to the formation of resveratrol [251].

### 9.3. Pharmacokinetics: Bioavailability, Metabolism, and Absorption

Rutin has been studied for its bioavailability and metabolism. The research indicates that rutin supplementation can elevate plasma flavonoids without any adverse effects on liver function [252]. Rutin is metabolized by the gut microbiota and absorbed as conjugated metabolites, making it bioavailable [253]. In rats, rutin is absorbed more slowly than its aglycone, quercetin, due to cecal microflora hydrolysis [254]. Studies have identified quercetin sulfates and glucuronides as the major metabolites of both rutin and quercetin in rats, influencing their bioavailability [255]. In rabbits, the metabolic fate of rutin and quercetin has been linked to the urinary excretion of its degradation products, including 3,4‐dihydroxyphenylacetic acid [256]. Several studies have therefore explored the methods to improve the bioavailability of rutin. These studies collectively suggest promising strategies to improve its dissolution rate, permeability, and bioavailability, such as the use of nanoemulsions [257], encapsulation [258], and chitosan nanoparticles [259]. For example, complexation with 2-hydroxypropyl-β-cyclodextrin increased rutin's solubility, dissolution rate, and oral bioavailability [260].

The absolute bioavailability of hyperin is approximately 26%, indicating a moderate absorption rate in the body [261]. Hyperin has showed to undergo rapid metabolism in rats, leading to the formation of 33 metabolites identified in the plasma, urine, and feces, with quercetin and dihydroquercetin being the most prominent ones [262]. Studies suggest that hyperin exhibits bimodal absorption after oral administration in rats, possibly owing to hepatoenteric circulation or absorption by different parts of the intestine [263]. Methylation, sulfation, and glucuronidation are the major metabolic pathways of hyperin in *vivo* [264]. Human intestinal bacteria can further metabolize hyperin, producing metabolites such as benzoic acid, quercetin, and 3,4-dihydroxyphenylacetic acid [265]. Pharmacokinetic studies recommend its intraperitoneal or intravenous administration for improved efficacy due to low oral bioavailability [263].

Isoquercitrin has been found to have higher bioavailability than quercetin [266]. Pure isoquercitrin can be obtained on a large scale through enzymatic rutin hydrolysis with α-l-rhamnosidase. Although small amounts of intact isoquercitrin can be detected in plasma and tissues after oral administration, it is extensively metabolized in the intestine and liver [267]. Its metabolites were identified as quercetin, acetylated isoquercitrin, dehydroxylated isoquercitrin, hydroxylated quercetin, and hydroxymethylated quercetin [268]. Enzymatically modified isoquercitrin is more easily absorbed compared to other quercetin glycosides or aglycones after oral administration in rats. This enhanced absorption contributes to its bioavailability and therapeutic efficacy [269]. Isoquercitrin undergoes biotransformation in the intestine and liver, involving deglycosylation followed by forming conjugated and methylated derivatives of quercetin or degradation to phenolic acids and carbon compounds [267]. The bioavailability of isoquercitrin can be further enhanced using dietary isoquercitrin-γ-cyclodextrin molecular inclusion complex, as has been demonstrated in both rats and humans [270].

The oral bioavailability of resveratrol is almost zero because of its rapid and extensive metabolism, leading to the formation of various metabolites [271]. The research on absorption, metabolism, and bioavailability of resveratrol has been conducted using different models such as in *vitro*, *ex vivo*, and in *vivo* studies. These studies have showed that resveratrol is absorbed and metabolized in the body. Approximately, 75% of this polyphenol is excreted via various pathways after ingestion [272]. Resveratrol metabolism involves the formation of glucuronide and sulfate conjugates during absorption, which decreases the levels of free trans-resveratrol circulating in the body [273]. Additionally, in the rat small intestine, resveratrol is absorbed and metabolized through its conversion to resveratrol glucuronide [274]. Despite its low bioavailability, resveratrol is well-tolerated and has no marked toxicity in humans [275]. The bioavailability of resveratrol can be improved by its dimethyl ether analog, pterostilbene, which has greater bioavailability as shown by increased total plasma levels [276]. Other methods, such as nanoencapsulation and coadministration with piperine, have also showed promise for enhancing resveratrol's bioavailability [277].

### 9.4. Toxicity and Side Effects

Rutin, isoquercitrin, hyperin, and resveratrol are generally considered safe in lower doses but may have a different safety profile depending on dosage and exposure duration. Rutin is relatively nontoxic at lower doses, as demonstrated by acute toxicity studies in mice that yielded an LD50 of approximately 1510 mg/kg in male animals and 1490 mg/kg in female animals [278]. In rats, the administration of dietary concentrations up to 1% (542.4 mg/kg/day for males and 674 mg/kg/day for females) over 52 weeks did not produce any adverse effects [278]. On the other hand, large doses of rutin were shown to reduce erythrocytic parameters and attenuate weight gain in males [279]. Isoquercitrin is safe in humans, with an acceptable daily intake of 5.4 mg/kg/day, although higher doses induce benign chromaturia in rats [279]. Another similar compound with higher bioavailability, the enzymatically modified isoquercitrin, is also considered a safe food additive [280]. In rats, the no-observed adverse effect level is known to be 300 mg/kg/day, while doses of resveratrol higher than 3000 mg/kg/day will induce renal toxicity and other adverse effects [281]. In humans, up to 1000 mg/day is well tolerated, but gastrointestinal symptoms have been reported when the dose exceeds 2500–5000 mg [282]. Likewise, hyperin at high dosage (1000 mg/kg/day) can cause renal toxicity that results from its accumulation in the kidneys, but this damage can be reversed after stopping it [283]. In contrast, chronic toxicity studies with lower doses of hyperin (30–175 mg/kg/day) in rats reported no significant adverse effects on behavior, food intake, or weight gain [284].

## 10. Conclusion and Perspectives

In conclusion, *Z*. *lotus* contains bioactive compounds such as rutin, isoquercitrin, hyperin, and resveratrol, which exhibit remarkable potential in alleviating metabolic syndrome and its associated conditions—obesity, diabetes, hypertension, and dyslipidemia. The therapeutic effects are mediated through several critical mechanisms. *Z. lotus* acts as a regulatory agent for obesity by regulating lipid metabolism through the activation of PPARs and promoting lipid oxidation through AMPK activation. It enhances insulin sensitivity by upregulation GLUT4 expression and decreases postprandial hyperglycemia by suppressing α-glucosidase activity in diabetes. In hypertension, it restores endothelial function through the decrease of oxidative stress and an increase in NO bioavailability, consequently causing vasodilation. In terms of dyslipidemia, it reduces TC and TG levels through the inhibition of important enzymes such as HMG-CoA reductase as well as the modification of lipid metabolism pathways.

Moreover, the plant's antioxidant properties counteract reactive oxygen species, and its anti-inflammatory properties inhibit proinflammatory cytokines, which play a pivotal role in the prevention of the development of cardiovascular and metabolic complications. This holistic targeting of various pathways associated with metabolic syndrome and its associated diseases positions *Z. lotus* as a potential source of novel therapeutic agents that may have clinical relevance. These results are consistent with the traditional uses of *Z. lotus* in regions of the Middle East and Africa as a remedy for managing symptoms of diabetes, hypertension, and metabolic disorders, substantiating the therapeutic power of this plant.

However, while these traditional uses form a vast base, their effectiveness and safety need further experimental and clinical studies to validate them. Although toxicity studies report an overall good safety profile for *Z. lotus*, these data are predominantly focused on acute toxicity tested in animal models, making the long-term safety and human relevance uncertain. Furthermore, this also underlines the ongoing key limitation of a dearth of adequately powered clinical trials, yielding limited data in terms of standardized dosages, formulations, and more varied patient cohorts. The challenges still lie in discovering more effective strategies to improve bioavailability. These gaps need to be overcome in future research, which should ideally include well-designed in vivo “proof-of-concept” experiments, elucidating pharmacological effects, pharmacokinetics, pharmacodynamics, and any potential interactions in a system biology context. For dosages and formulations to be equivalent, standardization is the key; this will lead to consistent efficacy and safety, allowing for clinical effects to be optimized across patient populations. This review has raised several important issues that should be discussed in the future to promote their application and development.

## Figures and Tables

**Table 1:** Ethnomedicinal usage of different parts of *Z*. *lotus* (L.) in various countries.

Country	Plant part used	Medicinal uses	References
Morocco	Whole plant	Smallpox, measles, boils, sores, abscesses, digestive and respiratory problems, circulatory and urinary complaints, pyelonephritis, diabetes, symptoms of COVID-19, urine retention, diuretic, renal, colic, kidney stones, urogenital, dermatological, cardiovascular diseases, rheumatism, typhoid infection, ear disorders, and skin problems	[34–45]
	Stems	Snake bites	[34]
	Leaves	Haircare, renal problems, gastric lavage, liver diseases, helminthiasis, colds, heart diseases, diabetes, urolithiasis, hypertension, hair loss, abscesses, sedative, diarrhea, cardiac failure, gastralgia, boils, soars, urinary infections, stomach pain, kidney stones, pneumonia, ulcers, and anorexia	[10, 11, 34, 46–63]
	Roots	Diabetes, stomach pains, diarrhea, kidney stones, jaundice, pulmonary and heart diseases, urolithiasis, intestinal pain, cough, bronchitis, and pyelonephritis	[46, 48, 54, 64–68]
	Fruits	Renal problems, cardiac ailments, pulmonary infection, hemostatic, colic, throat pains, pectoral pain, irritation, diabetes, hepatitis, immune system, depression, urolithiasis, kidney stones, stomach pain, fever, vomiting, renal pain, cystitis, pyelonephritis, cough, dyspnea, ulcers, diarrhea, and anorexia	[11, 37, 46–48, 50, 51, 53, 56, 60–62, 65, 67, 69–71]
	Seeds	Kidney problems, digestion problems, intestinal comfort, bloating, and diabetes	[72–74]
	Bark	Constipation, tachycardia, hemorrhoids, liver edema, stomachache, and pyelonephritis	[67, 75].

Algeria	Whole plant	Colorectal lung and breast cancers, bladder inflammation, prostate kidney and lung infections, jaundice, irritation, and lung affections	[76–79]
	Leaves	Eczema, cicatrization, inflammations, skin problems, breast cancer, eye problems, fever, stomach acidity, hypertension, heartburn, constipation, catarrhal, diabetes, and urinary infections	[80–89]
	Roots	Diabetes, toothache, gastrointestinal and liver disorders, inflammations, irritation and pectoral pain, pulmonary affections, jaundice, and stomachache	[68, 90–94]
	Fruits	Diabetes, respiratory problems, sedative, pectoral pain, emollient, defensive hedge, urine retention, inflammations, hypertension, and stomach acidity	[83, 89, 95]

Nigeria	Whole plant	Cancer	[96]

Mauritania	Leaves	Fever, diabetes, skin infections, toothache, hypertension, abdominal pain, and kidney problems	[97]

Cameroun	Whole plant	Cough, tired, and food poisoning	[98]

Mauritius	Fruits	Asthma, ulcers, and hemorrhoids	[99]

Palestine	Leaves	Diarrhea	[100]

Jordan	Fruits	Cough and measles	[101]

**Table 2:** Major bioactive compounds identified in different parts of *Z*. *lotus* (L.).

Major compounds	Chemical constituents	Plant part	References
Flavonoids (flavonols, dihydrochalcones, flavones, and flavanones)	Myricetin rutinoside	Leaf and branch	[13, 14, 102–105]
	Glycoside quercetin		
	Quercetin 3-*O*-rutinoside (rutin)		
	Quercetin-3-β-*D*-glucoside (isoquercitrin)		
	Quercetin-3-galactoside (hyperoside or hyperin)		
	Quercetin-3-*O*-(2,6-di-*O*-rhamnosyl-glucoside)-7-*O*-rhamnoside		
	Quercetin-3-*O*-(2,6-di-*O*-rhamnosyl-glucoside)		
	Quercetin-3-*O*-(2,6-di-*O*-rhamnosyl glucoside)-7-*O*-glucuronide		
	Glycoside kaempferol:		
	Kaempferol-3-*O*-(2,6-di-*O*-rhamnosyl-glucoside)		
	Kaempferol-O-hexoside		
	Kaempferol-3-*O*-rutinoside (nicotiflorin)		
	Kaempferol-3-*O*-(6-*O*-rhamnosyl-glucoside)		
	Phloretin-di-*C*-hexoside	Leaf, root bark	
	Apigenin		
	Apigenin-*O*-hexoside-*O*-deoxyhexoside		
	Catechin		
	Epicatechin		
	Epicatechin gallate		
	Naringin		
	Isorhamnetin	Fruit	[14, 15, 105–107]
	Rutin		
	Hyperoside		
	Apigenin		
	Luteolin		
	Luteolin-7-*O*-glucoside		
	Apigenin 7-*O*-glucoside		
	Quercetin 3-*O*-rhamnoside-7-*O*-glucoside		
	Diosmin		
	Cirsiliol		
	Catechin		
	Phloretin-3′,5′-di-glucoside		
	Naringenin		
	Eriodictyol		
	Eriodictyol glycoside derivatives:	Fruit	[14, 15, 105–107]
	Eriodictyol-*O*-hexoside		
	Eriodictyol-*O*-pentoside		
	Eriodictyol-*O*-deoxyhexoside		
	Quercetin-3-*O*-galactoside	Arial part	[108, 109]
	Quercetin-3-*O*-rhamnoside		
	Rutin		
	Chamaejasmin	Seeds	[110]
	Glucoliquiritin apioside 5,6,7,8,3′,4′,5′-heptamethoxy flavanone robustaside D		
	Kaempferol-3-*O*-robinobioside		
	Gallocatechin		
	Luteolin 7-(6‴′-acetylallosyl-(1- > 3)-glucosyl-(1- > 2)-glucoside apigenin 7-methyl ether 5-(6″-malonylglucoside)		

Stilbenes	Resveratrol	Fruit	[14]

Phenolic acids	*p*-Hydroxybenzoic acid	Fruit	[15, 106, 111, 112]
	*p*-coumaric acid		
	Benzoic acid		
	Sinapic acid		
	*p*-coumaroyl glucose		
	Cinnamic acid derivatives		
	Galloyl shikimic acid		
	Trans-ferulic acid		
	Rosmarinic acid		
	Syringic acid		
	Protocatechuic acid		
	Gallic acid		
	Quinic acid		
	Caffeic acid derivatives		
	Gentisic acid	Leaf	[13, 14, 113]
	Fumaric acid		
	Gallic acid		
	Chlorogenic acid		
	Caffeic acid		
	Vanillic acid		
	p-coumaric acid	Leaf	[13, 14, 113]
	Benzoic acid		
	P-hydroxybenzoic acid		

Alkaloids (cyclopeptide alkaloids)	Lotusine A	Root bark	[114–116]
	Lotusine B		
	Lotusine C		
	Lotusine D		
	Lotusine E		
	Lotusine F		
	Lotusine G		
	Lotusanine A	Aerial part	[117]
	Lotusanine B		
	Sanjoinenine		
	Sanjoinine F		
	Frangufoline		

Saponins (dammarane type)	Jujuboside A	Root bark	[118, 119]
	Jujuboside C		
	Lotoside I		
	Lotoside II		
	Jujuboside B	Leaf	[120]
	Jujubasaponine IV		
	Jujubogenin glycosides		

**Table 3:** Summary of the key mechanisms targeted by *Z*. *lotus* (L.) bioactive compounds.

Bioactive compound	Mechanisms of action	Impact on metabolism	Impact on vascular function
Rutin	- Inhibition of ACE - Activation of insulin signaling (IR kinase activity, GLUT4 translocation) - Inhibition of α-amylase and α-glucosidase - Reduces the expression of G-6-Pase - Decreases HMG-CoA - Inhibits NOD-like receptor pyrin domain inflammasome pathways - Reduces TNF-α and IFNγ - Suppresses proinflammatory markers (TNF-α, MCP-1, IFNγ, IL-6, IL-2, and NF-κB) - Suppressing the expression of NADPH oxidase 4.	- Reduces blood glucose levels - Improves insulin sensitivity - Reduces dyslipidemia (TC, TG, and LDL-C) - Improved LPL and LCAT - Reduces FBG, HbA1c	- Endothelium-dependent vasorelaxation through NO-guanylyl cyclase pathway, ATP-sensitive K+ channel activation - Recovering NO generation - Improves vascular reactivity and baroreflex sensitivity

Hyperin	- Inhibition of α-glucosidase - Inhibits α-amylase - Upregulating the expression of PPARγ - Inhibits NF-κB	- Reduces blood glucose levels and plasma lipids (TC, TG, and LDL-C) - Improves insulin sensitivity - Suppresses inflammation	-Endothelium-dependent and independent vasodilation through KCa channels and NO - Increased nitric oxide bioavailability - Suppression of chymase (Ang-II-forming enzyme) - Reduces peripheral vascular resistance and coronary vessel medial thickness

Isoquercitrin	- Inhibition of α-amylase - Modulation of AMP-activated protein kinase (AMPK) - Regulation of insulin signaling genes (IR, IRS-1, IRS-2, and PKB) - Inhibition of Na+/K+-ATPase activity - Attenuates NF-κB and TNF-α expression	- Improves blood glucose control - Enhances insulin secretion - Reduces dyslipidemia - Inhibits adipogenesis. - Suppression of oxidative stress, inflammation, and mitochondrial dysfunction	- Endothelium-dependent vasodilation - Increase in nitric oxide and hydrogen sulfide in endothelial cells - Bioavailability enhancement of bradykinin and prostaglandin I_2_ - Reduction in vascular resistance - Protects endothelial cells by upregulating antiapoptotic proteins (Bcl-2) and downregulating proapoptotic proteins (p53, Bax, and caspase 3) - Exhibits potent diuretic effects by increasing Na+ excretion while maintaining K+ balance

Resveratrol	- Activation of SIRT1 and AMPK - Modulation of PPARγ and inflammatory pathways - Inhibition of α-glucosidase and lipid metabolism enzymes	- Improves FBG and HbA1c and insulin sensitivity - Reduces glucose and lipid levels - Decreases adipogenesis	- Endothelial NO generation via eNOS activation - Vasodilation through improved NO bioavailability - Suppression of Gi protein signaling in vascular smooth muscle

## Data Availability

Data sharing is not applicable to this article as no new data were created or analyzed in this study.

## Nomenclature

ACE: Angiotensin-converting enzyme
AMPK: AMP-activated protein kinase
BAT: Brown adipose tissue
GLUT4: Glucose transporter 4
HDL: High-density lipoprotein
HFD: High-fat diet
HMG Co-A: Hydroxy-methylglutaryl-CoA reductase
IC_50_: Half-maximal inhibitory concentration
IFNγ: Interferon gamma
LDL: Low-density lipoprotein
NOS: Nitric oxide synthase
SIRT1: Activated sirtuin 1
PPARs: Peroxisome proliferator–activated receptors
SREBP-1: Sterol regulatory element-binding transcription Factor 1
TC: Total cholesterol
TG: Triglycerides
TNF-α: Tumor necrosis factor
WAT: White adipose tissue
STZ: Streptozotocin
FBG: Fasting blood glucose
HbA1c: Glycated hemoglobin
PPARγ: Peroxisome proliferator–activated receptor *γ*
IR: Insulin receptor
PKB: Protein kinase B
HepG2: Human liver cancer cell line
G-6-Pase: Glucose-6-phosphatase
NF-κB: Nuclear factor
FAS: Fatty acid synthase
C/EBP: CCAAT/enhancer-binding proteins
ACC1: Acetyl-CoA carboxylase 1
FIAF: Fasting-induced adipose factor
L-NAME: Nω-nitro-L-arginine methyl ester
SHR: Spontaneously hypertensive rats
Ang-II: Angiotensin II
3T3-L1: Swiss 3T3 cell line, preadipocyte Clone 1
LD50: The median lethal dose
